# Artificial intelligence in medical education and the meaning of interaction with natural intelligence – an interdisciplinary approach

**DOI:** 10.3205/zma001352

**Published:** 2020-11-16

**Authors:** Johannes Lang, Holger Repp

**Affiliations:** 1 Justus-Liebig-University Gießen, Medical Faculty, Dean's Office, Division for Study and Teaching, Gießen, Germany

**Keywords:** artificial intelligence, interdisciplinary research, interdisciplinary learning, evaluation, teaching

## Abstract

**Introduction: **The practice of medicine is characterized by decision making in which digital techniques can provide good support. In this context, artificial intelligence (AI) is becoming increasingly important. The challenge for physicians, however, is to maintain an overview of the potential applications and usefulness of AI in order to be able to apply it efficiently and safely in their work. Therefore, appropriate skills must be imparted during the course of medical studies so that future practitioners can meet this requirement.

**Project description: **The interdisciplinary research-related teaching and learning project “(Natural) Science and Technology in Medicine – NWTmed” brings together students at the Justus-Liebig-University Giessen (JLU) from the fields of medicine and other (natural) scientific disciplines in structured courses with the aim of thinking, learning, and working in an interdisciplinary and research-oriented manner already during their medical education. With the involvement of local researchers, a “multi-disciplinary” seminar on the basic premises, methods, and applications of AI was established.

**Results: **The participants of the course came from a wide variety of fields of study, which promoted an interdisciplinary exchange and animated discussions. A gain in knowledge and an increase in interest in the topic of AI was noted in the evaluations, and a willingness on the part of the students to pursue further independent study was also expressed.

**Discussion and conclusion: **The topic of AI and its relevance to the field of medicine is not yet sufficiently represented in medical education. It will require integration in the curriculum and performance evaluations as well as interdisciplinary and research-related teaching formats.

## Zusammenfassung

**Einleitung:** Die ärztliche Arbeit ist geprägt vom Treffen von Entscheidungen, bei welchen digitale Techniken eine gute Unterstützung bieten können. In diesem Kontext erlangt Künstliche Intelligenz (KI) eine zunehmende Bedeutung. Die Herausforderung für die Ärzt*innen ist allerdings, fortwährend den Überblick zu Einsatzmöglichkeiten und Sinnhaftigkeit bei der Nutzung von KI zu behalten, um sie bei ihrer Tätigkeit effizient und sicher anwenden zu können. Daher müssen bereits während des Studiums entsprechende Kompetenzen vermittelt werden, damit die zukünftigen Ärzt*innen diesem Anspruch gerecht werden können.

**Projektbeschreibung:** Das interdisziplinäre forschungsnahe Lehr- und Lernprojekt „(Natur)Wissenschaft und Technik in der Medizin – NWTmed“ bringt Studierende an der Justus-Liebig-Universität Gießen (JLU) aus der Medizin und naturwissenschaftlichen Fachbereichen strukturiert in Lehrveranstaltungen zusammen, mit dem Ziel bereits während des Studiums interdisziplinär und forschungsnah zu denken, zu lernen und zu arbeiten. Unter Einbezug lokaler Forscher*innen konnte ein „multi“disziplinäres Seminar zu Grundlagen, Methoden und Anwendungen der KI etabliert werden.

**Ergebnisse:** Die Teilnehmer*innen des Lehrangebots setzten sich aus unterschiedlichsten Studienbereichen zusammen, was einen interdisziplinären Austausch und eine angeregte Diskussion beförderte. Ein Zuwachs an Kenntnissen und die Steigerung des Interesses an der Thematik KI wurde in den Evaluationen festgestellt und die Bereitschaft zu weiterführendem Engagement von Seiten der Studierenden auch im Selbststudium wurde geäußert.

**Diskussion und Schlussfolgerung: **Das Themenfeld KI und dessen Relevanz ist im Medizinstudium noch nicht hinreichend abgebildet. Es bedarf einer curricularen Einbindung und einer Repräsentanz auch in Leistungsnachweisen sowie interdisziplinärer und forschungsnaher Lehrformate.

## Introduction

An important element of medical work is the making of decisions, and the time factor often plays an essential and limiting role. With the rapidly advancing availability and further development of digital technologies and devices and the direct online access to a wide range of information sources, it is hoped not only that the speed of the decision-making process and medical actions will increase but also that the quality of care will improve. However, a major challenge for practitioners is to maintain an overview of the digital possibilities and tools in order to be able to use them efficiently in their work, for which special medical training is under discussion [1]. Therefore, it is important to teach these competencies in a structured way during medical studies so that future physicians can meet this requirement [2], [3]. In particular, the use and further development of AI is highly relevant to this training.

## Description

The interdisciplinary research-related teaching and learning project "(Natural) Science and Technology in Medicine – SciTecMed (NWTmed in German)" was launched during the winter semester of 2017/18 at the Department of Medicine of the JLU Giessen, and two more (natural) scientific faculties were added for the winter semester of 2018/19. SciTecMed brings together students from medicine and the natural sciences in a structured way in courses designed to promote thinking, learning, and working in an interdisciplinary and research-oriented manner [4]. The course contents are based on the local research priorities and are presented by the researchers themselves from their everyday work. This allows students to experience research not only in an abstract but also in an authentic and personal way and to reflect upon and discuss it in an interdisciplinary atmosphere. Since the winter semester of 2018/19, experienced AI users [5], [6] have been offering a multi-disciplinary seminar as an elective (clinical study section for medical students; 2 CP for science students and 3 CP with separate project work) on the basics, methods, and applications of AI with 11 sessions (each 120 min.) in the form of seminars, guided discussions, and demonstration and concrete development of algorithms. The content and the organization of the events were coordinated by the SciTecMed/NWTmed project [7].

## Results

The participants (47 total) were students from the fields of biology, chemistry, history, law, materials science, mathematics, medicine, medical informatics, and physics. An initial survey taken during the summer semester of 2019 revealed the following interests: an overview of AI applications in different areas and ongoing projects; current and future applications in medicine; error assessment of AI results and technical insight into AI processes; use in economics and implementation in products; ethical aspects and influence on social change. These interests were covered in the seminars, which had as main topics: AI in personal everyday life; AI examples in medicine; scientific use and potentials of AI; construction of neural networks and principles of deep learning; expansion of basic knowledge of statistics; "NeuroTronics", with parallels from biology and electronics; radiological applications; mathematical description in AI procedures; ethical aspects; guided programming and practical implementation. Critical aspects of ethics and responsibility, limitations, and possible dangers were handled in discussions. The final evaluation, which was conducted with teaching evaluation sheets provided by the university, showed that the majority of the participants (n=10; low participation due to concurrent examinations) would have liked to have explored the topics in greater depth and to have had more time. Self-assessed knowledge rose from an initial 2.3 to 3.8 on the Likert scale (1-5). Interest in the topic was initially 4.3 and increased to 4.8. The approaches within different disciplines, including theoretical, practical, and ethical considerations, the respectful atmosphere, and the interdisciplinary group discussions were particularly welcomed. 

## Discussion

The interdisciplinary approach is very sustainable and motivating from the perspective of both students and teachers but requires additional coordination and adjustments. Scientific expertise combined with sound application competence taken directly from scientific and technical research helps the participants to make a critical assessment on the one hand and promotes creative openness on the other, which goes well beyond dogmatic training of technological competence. Although the involvement of motivated participants in the compulsory elective courses is very lively, students who are less aware of the topic should also be confronted with a scientifically in-depth discussion; they are best aligned with a broader series on digitalization topics. Although the importance of AI in medicine is increasing [8], it seems that many medical students lack awareness of this and therefore miss out on taking their own initiative. There are also some instances of restraint, which may be due to the own perception of a lack of basic knowledge. This must be counteracted with easily accessible course offerings and by presenting the relevance of the subject matter, also to other disciplines. Transferability and adaptation to other sites and locations is conceivable and desirable. Scaling up towards large semester cohorts should be carried out in parallel in discussion-compatible groups.

## Conclusion

The interest of medical students in AI could be significantly increased by a structured integration in the curriculum and also by an increased presence in the national competence-oriented learning objective catalogue (NKLM) [2]. In the interdisciplinary approach, it is advisable to take into account differences in the level of prior knowledge of the participating students from the various study programmes and to actively incorporate existing student expertise into teaching.

## Acknowledgements

The authors would like to thank PD Dr. Olena Linnyk for her initiative in offering an AI course, Dr. Martin Obert for his expertise in big data management, and both of them for their openness to the teaching project SciTecMed/NWTmed.

The work is supported by central and decentralized QSL funds of the JLU Giessen as well as by funds from the study structure program of the State of Hesse.

## Competing interests

The authors declare that they have no competing interests. 
